# Cullin E3 Ligases and Their Rewiring by Viral Factors

**DOI:** 10.3390/biom4040897

**Published:** 2014-10-13

**Authors:** Cathal Mahon, Nevan J. Krogan, Charles S. Craik, Elah Pick

**Affiliations:** 1 Department of Pharmaceutical Chemistry, University of California San Francisco, San Francisco, CA 94158-2530, USA; E-Mails: cathal.mahon@ucsf.edu (C.M.); charles.craik@ucsf.edu (C.S.C.); 2 Department of Cellular and Molecular Pharmacology, University of California San Francisco, San Francisco, CA 94158-2530, USA; E-Mail: Nevan.Krogan@ucsf.edu; 3 Department of Biology and Environment, Faculty of Natural Sciences, University of Haifa at Oranim, Tivon 3003500, Israel

**Keywords:** ubiquitin, proteasome, E3, cullin, F-box protein, virus, cullin-RING ligase

## Abstract

The ability of viruses to subvert host pathways is central in disease pathogenesis. Over the past decade, a critical role for the Ubiquitin Proteasome System (UPS) in counteracting host immune factors during viral infection has emerged. This counteraction is commonly achieved by the expression of viral proteins capable of sequestering host ubiquitin E3 ligases and their regulators. In particular, many viruses hijack members of the Cullin-RING E3 Ligase (CRL) family. Viruses interact in many ways with CRLs in order to impact their ligase activity; one key recurring interaction involves re-directing CRL complexes to degrade host targets that are otherwise not degraded within host cells. Removal of host immune factors by this mechanism creates a more amenable cellular environment for viral propagation. To date, a small number of target host factors have been identified, many of which are degraded via a CRL-proteasome pathway. Substantial effort within the field is ongoing to uncover the identities of further host proteins targeted in this fashion and the underlying mechanisms driving their turnover by the UPS. Elucidation of these targets and mechanisms will provide appealing anti-viral therapeutic opportunities. This review is focused on the many methods used by viruses to perturb host CRLs, focusing on substrate sequestration and viral regulation of E3 activity.

## 1. Introduction

Successful viral replication requires modifying and hijacking key cellular pathways within host cells. In particular, viruses must overcome host immune responses to successfully survive and propagate. To achieve this, viruses regularly usurp the host cells degradation machinery. The predominant degradation apparatus within host cells is the Ubiquitin-Proteasome System (UPS), which is responsible for targeted protein degradation. The UPS regulates proteins involved in most fundamental cellular processes including transcription, translation, DNA repair and the cell cycle. Critically, the UPS can rapidly respond to environmental changes such as viral infection [1]. In this system, a cascade of three enzymes activate (E1); conjugate (E2); and ligate (E3) a polypeptide of ubiquitin to protein substrate targets, resulting mostly (though critically not always) in their elimination from the cell by the proteasome [1,2].

Components of the UPS are tightly regulated by exquisite mechanisms involving cycles of precisely timed E3 activation/inhibition and dynamic E3 protein complex remodeling. Substrate targeting and degradation is a highly selective process and commonly involves an upstream posttranslational pathway “tagging” a substrate marking it for degradation. The tagged substrate then interacts (in)directly with a specific E3, (one out of an expanding number of ~600 monomeric and multimeric enzymes), driving the ubiquitin transfer [3]. The resulting ubiquitinated substrate is then shuttled by chaperones to the proteasome and ultimately the polypeptide is hydrolyzed and degraded [4].

Viruses have co-evolved to utilize the host UPS for their own requirements in many aspects of their life cycle, including driving virus egress from the host cell [5], increasing viral replication [6], altering host cell cycle status [7] and restricting host-defense mechanisms [8].The regulation of the UPS is an Achilles' heel within host defense systems, which many pathogens including viruses take advantage of to counteract host defenses [9,10,11].

A pivotal mechanism in hijacking the UPS is through host–viral interactions with ubiquitin E3 ligases (and their regulators)—as these enzyme complexes are directly responsible for substrate selection and turnover. There are a number of well-established examples, one of the best defined being the papillomavirus E6 protein. This viral protein binds cellular E3s redirecting them to degrade host proteins including p53, driving oncogenesis in infected cells [12].

**Figure 1:**
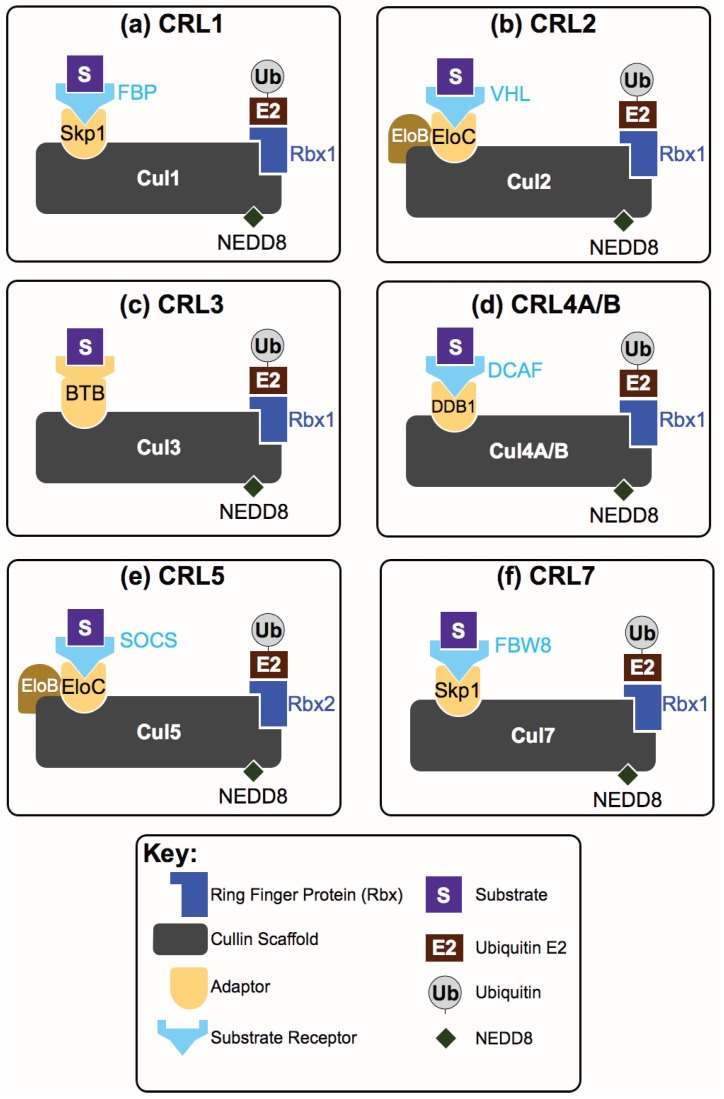
Modular Cullin RING ligase (CRL) complexes. CRL complexes include one out of seven cullins (Cul1, 2, 3, 4a, 4b, 5, 7) as a scaffold subunit. Each cullin recruits an Rbx protein onto which an E2 conjugated with a single ubiquitin can load (see Figure 2). All CRL complexes have an adaptor protein that links the substrate receptor to the cullin placing the substrate and the E2 in close proximity. In the case of CRL3 complexes, the adaptor and SR are within the same (BTB) polypeptide.

**Figure 2:**
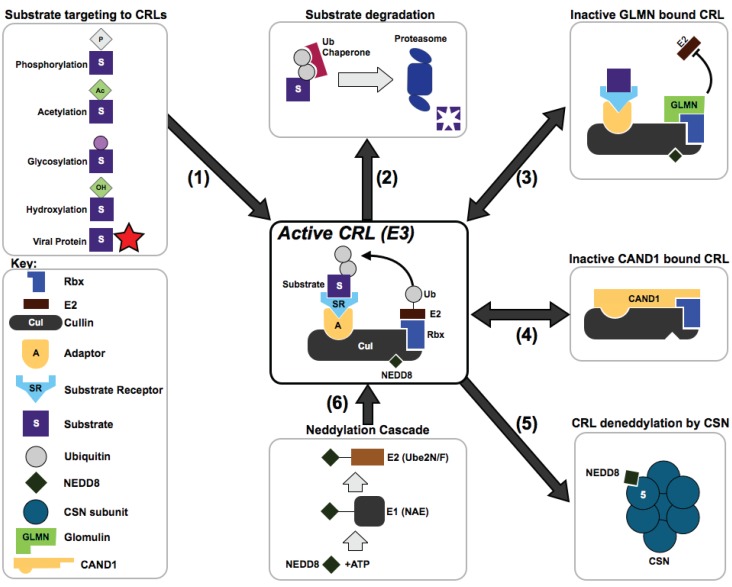
Cullin RING Ligase Activity and Regulation. CRLs are active when the cullin subunit is neddylated, and bound to an adaptor molecule (A), and a substrate receptor (SR), that recognizes a specific substrate (S). (1) Host substrate proteins are directed to active CRL complexes by post-translation modifications (phosphorylation, acetylation, glycosylation and hydroxylation) or by binding to a viral protein; (2) Ubiquitination of substrates often (though not always) results in their degradation by the 26S proteasome; (3) and (4) CRL complexes exist in several conformations including inhibited/intermediate states bound by GLMN or CAND1; (5) Removal of NEDD8 by the CSN results in inactive conformations. In addition, the CSN is capable of non-enzymatic regulation of cullins [21,22]. (6) The activating NEDD8 moiety is covalently attached to cullins by a cascade of three enzymes.

The largest families of E3 ligases—the multisubunit Cullin-RING E3 Ligases (CRLs)—are responsible for up to 20% of all ubiquitinated substrates within cells [13]. In CRL complexes, the cullin subunit (Cul1-7) functions as a scaffold onto which (a) an adaptor subunit; (b) a substrate receptor (SR) and (c) a regulator of Cullins/RING box protein-1(ROC1/Rbx1) get loaded (Figure 1). This assembled complex functions to allow the conjugation of ubiquitin from a bound E2 to the target substrate. CRLs are activated and inhibited by a number of intertwined mechanisms. Activation occurs when the cullin scaffold subunit is post-translationally modified by the ubiquitin-homologous protein NEDD8 (Neural precursor cell expressed developmentally down-regulated protein 8)—termed neddylation, and conversely inactivation occurs through NEDD8 removal by hydrolysis (deneddylation) [14,15]. Other factors involved in CRL regulation include (a) the cullin-associated NEDD8-dissociated protein 1 (CAND1)—which locks CRLs in a non-substrate binding conformation and functions as an SR exchange factor [16], and (b) the E2 competitor/inhibitor Glomulin (GLMN)—which allows partial CRL complex formation while blocking the CRL–E2 interaction (Figure 2) [17].

Several key mechanisms specifically relating to viral exploitation of CRLs have been elegantly studied (see Table 1). These studies have shown that multiple viral proteins specifically target a large array of CRL complexes typically regulating their substrate targeting activity [18,19,20]. The aim of this review is to focus on how specific viruses rewire ubiquitination of CRL substrates and how these in turn impact on viral infection. We will first describe the CRL family of E3 ligases and its regulation. We will also provide a comprehensive overview of the latest findings on viral factors that regulate CRLs.

**Table 1:** Viral hijacking of host CRLs.

Virus	Viral Protein	UPS Factor Targeted	Viral Protein Function	Reference
**Group I**				
Ectromelia	4 × BTB-kelch type ^1^	CRL3	Form complexes with CRL3, substrates unknown. EVM150 inhibits NFκB independently of CRL3	[23,24]
VACV	Unknown	CRL3	Unknown	[25]
Adenovirus	E4ORF6 & EB155K	CRL2/5	Serotype specific degradation of p53, TOPBP1, MRE11, BLM, ATRX, integrin α3 & DNA ligase IV	[26,27,28,29,30,31,32]
HPV	E7	CRL2 & proteasome	Degradation of pRb	[33]
KSHV	LANA	CRL5	Acts as a substrate receptor for P53 and VHL degradation	[34,35,36]
	LANA	CRL1	Binds to FBW7 inhibiting ICN degradation; Interacts with GSK-3 and inhibits c-Myc degradation by CRL1	
	V-cyclin	CRL1	Causes phosphorylation of p27 which is then degraded by CRL1	
Myxoma virus	MT-5	CRL1	Drives degradation of p27 regulating cell cycle progression at G_0_/G_1_	[37]
AcMNPV	Lef7	CRL1	Functions as an F box protein, alters the host DNA damage response through an unknown mechanism	[38]
MuHV-4	ORF73	CRL5	Functions as a substrate receptor to degrade p65- inhibiting NFκB signaling	[39]
CELO virus	GAM1	CRL2/5	Drives SAE1 and SAE degradation	[40]
SV40	LTAg	CRL7	Inhibits CRL7 degradation of IRS-1	[41,42]
HSV1	ICPo	Proteasome and E2	Trans-activates host cellular genes through unknown mechanisms	[43]
EBV	BZLF	CRL2	Functions as a substrate adaptor driving p53 degradation	[44]
	BPFL1	CRLs	Acts as a deneddylase inactivating CRLs preventing the degradation of host cell cycle and DNA damage regulators (Cdc25A, CDT1, p21, and p27)	[45]
**Group II**				
TYLCSV	C2	CSN5	Inhibits CSN activity and deregulates CRL activity	[46]
MVM	Unknown	CRL4	Infection causes CRL4 driven degradation of host p21	[47]
FBNYV	Clink	SKP1	Contains a functional F box with no known biological role	[48]
**Group III**				
Rotavirus	NSP1	CRL1	Causes degradation of β-TRCP	[49]
**Group IV**				
HepE	ORF2	CRL1	Inhibits IκBa ubiquitination by CRL1	[50]
BNYVV	P25	CRL1	Probably interacts with host F box proteins	[51,52,53]
Polerovirus	P0	CRL1	Targets a yet undiscovered host factor involved in post-transcriptional gene silencing	[54]
**Group V**				
Paramyxoxy virus family	V proteins	CRL4	Degrades host STAT proteins— disabling host interferon response	[55,56]
RSV	NS1	CRL2	Degrades host STAT proteins—disabling host interferon response	[57]
Rift Valley Fever	NSs	CRL1	Binds to FBXO3 driving degradation of TFIIH subunit	[58,59]
**Group VI**				
HIV-1	Vif	CRL5	Acts as a substrate receptor driving degradation of A3 proteins	[60]
	Vpu	CRL1	Assembles in a β-TRCP-containing SCF complex driving CD4 and possibly BST2 degradation	[61]
	Vpr	CRL4	Prematurely activates SLX4 complex by driving Mus81 ubiquitination	[62]
HIV-2/SIV	Vpx	CRL4	Drives SAMHD1 degradation in specific cell types	[63,64,65]
**Group VII**				
HepB	HBx	CRL4	Causes genome instability through an unknown mechanism	[64,66]

* also includes mumps and para-influenzavirus; ^1^ Four ectromelia proteins: EVM018, EVM027, EVM150 & EVM167.

## 2. How Are CRL Complexes Formed?

CRLs are modular complexes that constitute the largest class of E3 ligases [67] and are represented by the archetypical Skp1-Cul1-F-box (SCF) complex [3,68] (Figure 1a). Within the SCF complex, Cul1 functions as a scaffold subunit that interacts with Rbx1 via its C-terminus, and with an adaptor known as S-phase kinase-associated protein-1 (Skp1) via its N-terminus. Skp1 in turn binds to an SR belonging to the family of F-box proteins (FBPs or FBXs). The FBP recruits one or more specific target substrates for poly-ubiquitination and often subsequent degradation (Figure 2) [68,69]. In human cells, the family of FBPs consists of ~70 proteins and it is assumed that each SR targets one or more unique substrates within cells [70].

Seven different cullin scaffold proteins exist in humans (Figure 1a–f), each serving as a building block for the assembly of many modular multi-subunit CRL complexes. Our knowledge of the broad range of functions of CRLs is constantly expanding with the identification of each new CRL conformation. A great deal of knowledge can be gained specifically if the CRLs regulatory “state” (*i.e.*, whether it is active/inactive) or if the identity of its substrate(s) are also known.

It is possible to subdivide CRLs into clusters according to the interaction mode between the cullin scaffold subunits and their adaptors (Figure 1). CRL3s are unique in that their SR module and adaptor protein are within a single polypeptide (broad complex, tramtrack, bric-a-brac {BTB} fold proteins) (Figure 1c) [71]. It is possible to divide the other six CRLs into three archetypes according to the recruited adaptor and SR: (1) CRLs containing a Skp1 adaptor and an FBP SR (Cul1 and Cul7) (Figure 1a,f); (2) CRLs containing an Elongin B/C heterodimer as an adaptor, which connects Cul2 and Cul5 CRLs (Figure 1b,e) with their respective SRs (called BC or Suppressor of Cytokine Signaling (SOCS) proteins); (3) The highly related Cul4A and Cul4B CRLs with DNA damage binding protein-1 (DDB1) as an adaptor and DDB1/Cul4A associated factor (DCAF) proteins as SRs [14] (Figure 1d). Interestingly, these three archetypes are highly conserved in evolution, and each one is represented across phyla, from budding yeast to human. In addition to these minimal archetypes, CRLs have been shown to contain many other cofactors—the roles of which are poorly understood (for example, the DDA1 cofactor with Cul4A/B [72,73]). Furthermore, CRLs are capable of forming homo- and possible hetero-multimers vastly expanding their conformational space [74,75,76]. This tunable modularity highlights CRLs as a supergroup of ubiquitin ligase E3 enzymes capable of exerting a broad impact on protein turnover in many biological processes within eukaryotes.

## 3. How are CRL Complexes Regulated Within Cells?

The regulation of cullins involves several alternate complex conformations. Activation occurs when the cullin subunit is covalently modified by NEDD8 (Figure 2, step 6) [77]. The neddylation cascade begins with the activation of NEDD8 by the E1 enzyme NAE1 (NEDD8 Activating Enzyme-1) (Figure 2). NEDD8 is then conjugated to the NEDD8 E2 enzyme UBE2F (Ubiquitin-Conjugating Enzyme E2) in the case of CRL5; UBE2M (Ubiquitin-Conjugating Enzyme E2M) for all other CRLs. Ligation of NEDD8 to the cullin subunit is facilitated by two E3 enzymes that work in concert—DCN1 (Defective in cullin Neddylation-1) and RBX1 [78,79]. A neddylated cullin is capable of associating with an adaptor and an SR that can recognize and recruit a specific substrate (Figure 2) [77]. Ubiquitination occurs through a direct transfer of ubiquitin from the ubiquitin bound-E2 enzyme to a specific lysine residue of the substrate via the formation of a covalent bond. Repeated ubiquitination cycles lead to the poly-ubiquitination of the substrate (Figure 2; [80]).

Although neddylation is thought of as an activating “on-switch” for CRL substrate ubiquitination, it seems that neddylation/deneddylation cycles are required for CRL activity implying a complex cycling balance is required [81]. Deneddylation is carried out by the eight-subunit COP9 signalosome (CSN) complex and depends on a unique catalytically active metal-binding MPN^+^ [(Mpr1p and PAD1p N-terminal)/JAMM (JAB1/MPN/Mov34 metalloenzyme)] motif, harbored in its catalytic subunit, CSN5 (Figure 2, step 5) [82,83,84]. Precisely why the hydrolysis of the small polypeptide NEDD8, requires a highly conserved and intricate 440kDa eight-member complex is not yet understood but remains an intriguing question within the field. It is indeed likely that the CSN has other non-isopeptidase functions and this is why it has been referred to as “more than a protease” [85]. The CSNs ability to stabilize SRs *in vivo* has been described in many organisms, and in most cases dissociation of the CSN from a CRL results in the loss of SR stability and SR auto-ubiquitination [16,81,86,87,88]. Supporting this are the recent findings that in addition to the catalytic activity of Csn5, other CSN subunits are involved in regulating CRLs through a non-enzymatic manner [21,22,89]. Structural and biochemical studies have shown a non-enzymatic protective effect of the CSN on SRs such as the DNA damage binding protein-2 (DDB2) and Cockayne syndrome A1 (CSA1) in CRL4-DDB2 and CRL4-CSA1 complexes, respectively [21]. Comparable results were also found in *Neurospora crassa*, in which the CSN complex stabilizes five different SRs *in vivo* [89]. This protection may be caused by a steric effect of CSN binding to the CRL thereby inhibiting neddylation [22].

Taken together, it is evident that the CSN affects different CRL complexes to varying extents. Specifically, the CSN binds to CRL family members with different affinities [21,22] and alters the stability of unique CRL complex conformations. Further work will hopefully elucidate the physiological implications of the diversity of CSN–CRL connections particularly during viral infection.

In order for a formed CRL complex to recycle, deneddylation is required. This allows the binding of CAND1 to the CRL. When bound by CAND1, a CRL can exchange or swap an already bound “adaptor-SR” pair with a new SR capable of targeting a different substrate (Figure 2, step 4) [90]. A further level of CRL regulation *in vivo* was observed with the discovery of GLMN. This previously misunderstood protein displays a much higher affinity for Rbx1 than Cdc34 (a CRL E2) resulting in the dissociation of E2-ubiquitin complexes from the CRL (Figure 2, Step 3) [90,91]. A GLMN-CRL complex cannot therefore ubiquitinate its substrate and is deemed to be in an inhibited conformation.

A combination of NEDD8, CSN, CAND1, GLMN and likely more undiscovered factors all combine to carefully regulate a CRLs activity *in vivo*. As cells go to such lengths to regulate this family of E3s, it is clear they play pivotal roles in many cellular processes. This provides invading pathogens with a large array of targetable CRL subunits and associated components in a wide range of conformations to subvert CRL activity and the host UPS.

## 4. How Do CRLs Select Their Substrates?

Great strides have been made over the past decade in defining CRL mechanisms. A key advance in this understanding came with the solving of a number of high-resolution CRL structures [21,90,92,93]. One of the most crucial steps to determine in CRL biology is the molecular mechanism of how CRLs recognize and select their substrates. Hindering these studies is the high level of difficulty in determining physiologically *bona fide* SR-substrate pairs. Recruitment of target substrates by CRLs is a highly coordinated process involving several complex steps—complicating many standard experimental approaches. Our current understanding proposes that the SR protein plays a critical role in bringing the substrate in close proximity to the ubiquitin-loaded E2. This results in substrate degradation by either an effective increase in the local concentration of substrate and E2, or by positioning of the substrate in a favorable orientation by the SR allowing ubiquitination [94]. Many SRs only recognize their cognate substrates when the substrate is post-translationally modified at a specific region/sequence referred to as a degron. Modifications of degrons found to date include: acetylation, phosphorylation, glycosylation and hydroxylation (Figure 2, step 1) [69,95,96]. Other steps involved in substrate recognition include SR dimerization [67], DNA binding [21], interactions with unique co-factors [92] and the presence of small molecules such as hormones [69]. The latter is highlighted in plants where auxin interacting with an FBP (TIR1) is responsible for recognition of target proteins [97]. It is likely that the variety of degron modifications and SR-substrate interaction modes are even wider and involve as yet unknown factors. Further structural and biochemical CRL studies will likely provide a clearer picture of substrate recognition. Of particular interest will be discerning how viruses create/modify degrons when hijacking CRLs.

Taken together, these steps allow for the expansion of the number (and targeting mechanisms) of possible substrates for each CRL and many more examples are likely to be uncovered. However, with our current understanding it is clear that viral hijacking of CRLs utilizes similar mechanisms to that used in host-CRL substrate targeting as well as uniquely evolved viral mechanisms to direct host factors for degradation as described below [20].

## 5. How Do Viruses Affect CRLs?

As CRLs are responsible for the turnover of ~20% of ubiquitinated proteins [13], it is not surprising that many viruses hijack or co-opt host CRLs by expressing proteins that physically interact with subunits of one or more CRL family members. A clear example of this is the Human Immunodeficiency Virus (HIV), which uses (at least) three of its 18 encoded proteins (Vif, Vpu and Vpr) for interactions with five CRL family members (CRLs 1, 2, 4A/B & 5). These HIV–CRL interactions predominantly result in the poly-ubiquitination (and degradation/re-localization) of host anti-viral factors [20,98]. Below, we review the range of viral mechanisms that have been found to manipulate CRLs, including those used by HIV. We also describe the effect of the viral–CRL interaction on any known substrates and on viral infectivity.

We used the “Baltimore classification” system for viruses, which includes seven groups according to nucleic acids type (*i.e.*, DNA, RNA) and strandedness (*i.e.*, single strand, double strand) [99]. We observed that viruses of all seven groups interact with CRL family members and in addition many groups interact with CRL regulators. A common outcome of these host-viral interactions is the disabling of host defense mechanisms.

### 5.1. Group-I (Double Stranded DNA Viruses)

It is well established that viruses from this group interact with multiple CRLs (or their regulators). The ectromelia (pox) virus (the causative agent of mousepox) encodes a number of BTB/Kelch proteins. As described above, BTB proteins function as both the adaptor and SR within CRL3 complexes (Figure 1c). How the ectromelia BTB–CRL interaction affects pox-viral replication remains uncharacterized, but may involve NFκB signaling although both ubiquitination dependent and independent effects have been reported [23,24]. The potential ability of such viruses to interfere with NFκB signaling, a key player in early host immune response signaling, deserves further study. In addition, ectromelia viruses also encode their own FBPs [23] which complex with host CRL1 complexes, unfortunately no clear effect on substrate degradation has yet been identified. Together this evidence provides the first glimpses into poxvirus ability to hijack CRL subunits involved in substrate selection. Further studies are needed to demonstrate how this promotes infection—identification of the substrates degraded in a viral-dependent manner will help decipher the significance of pox-CRL interactions.

Further interesting evidence of a Group-I virus targeting CRL3 was uncovered in a recent host genome-wide RNAi screen with Vaccinia virus (VACV, the smallpox vaccine). This screen successfully identified two CRL components: Cul3 and Rbx1 as being required for viral replication. This also points towards a role for CRL3 in driving replication of certain viruses [25]. Identification of the mechanism and affected substrates (if any) in these cases will help decipher the physiological role of CRL3-VACV interactions *in vivo*.

In addition to initial exciting CRL3 connections, Group-I viruses also have a well-established role in CRL biology—specifically in affecting CRL2 and CRL5 complexes (both belonging to the same CRL archetype). This is exemplified in Adenoviruses, (viruses responsible for causing respiratory tract infections). Adenoviruses express E4ORF6 proteins that can bind both CRL2 and CRL5 host complexes in a serotype dependent fashion [26]. A common function across all serotypes is the ability of E4ORF6 proteins to form part of a functional CRL complex resulting in the degradation of host factors [100]. One example is the degradation of host DNA Ligase IV—the only host-factor targeted by all strains of adenovirus [27,100]. Intracellular viral DNA genomes form concatemers via a DNA ligase IV dependent non-homologous end-joining (NHEJ) pathway. Concatamer DNA is thought not to be efficiently packaged into virions and is presumed to be a dead-end for viral propagation [100]. Therefore, degradation of DNA ligase IV by the E4ORF6-CRL complex prevents concatamer formation enhancing viral propagation.

**Figure 3:**
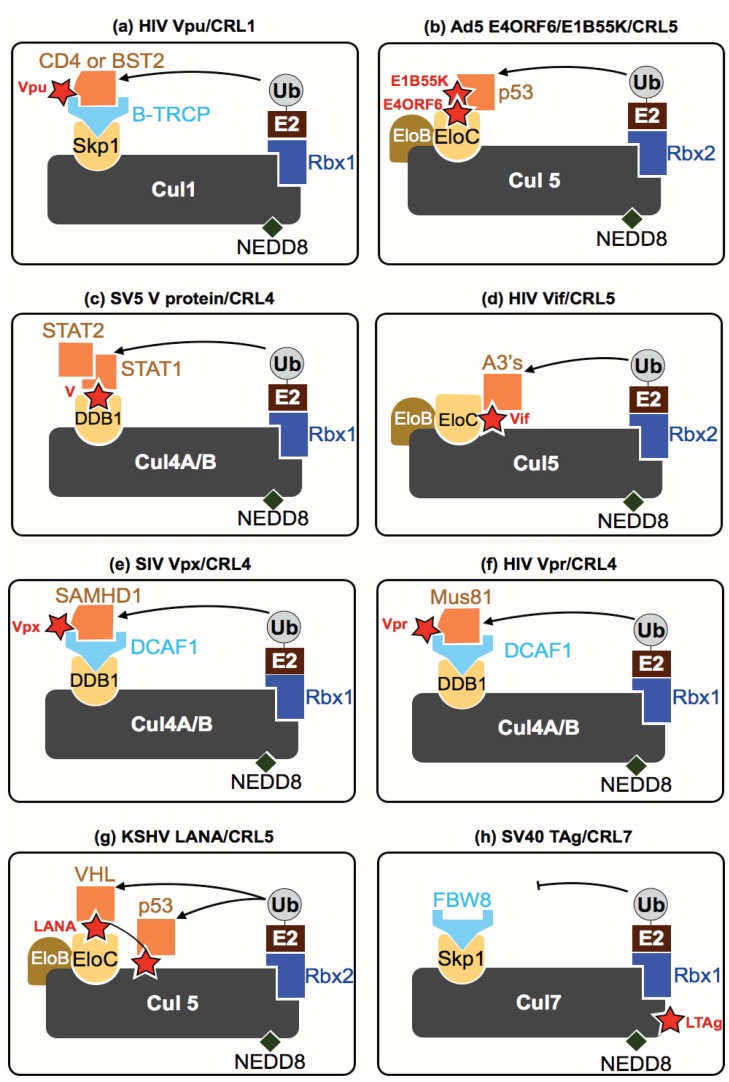
Key examples depicting viral hijacking of host Cullin RING ligases. Host CRLs are co-opted and hijacked by many viral proteins (depicted by a red star). (**a**) CRL1 hijacking by HIV Vpu; (**b**) CRL2 hijacking by adenovirus E1B55K and E4ORF6 proteins; (**c**) CRL4 hijacking by SV5 V protein; (**d**) CRL5 hijacking by HIV Vif; (**e**) CRL4 hijacking by SIV Vpx; (**f**) CRL4 hijacking by HIV Vpr; (**g**) CRL5 hijacking by KSHV LANA; (**h**) CRL7 inhibition by SV40 LTAg. Substrates degraded by specific complexes are shown in orange.

In another example, E4ORF6 of the Ad5 Adenovirus strain uses a second viral protein (E1B55K) as a cofactor that combines with E4ORF6 to form an active CRL5 complex driving p53 degradation (Figure 3b) [28,29]. Cellular infection by many viruses often causes p53 stabilization with cells—this increase in p53 levels can result in cell cycle arrest and premature apoptosis, both of which would have a drastic negative effect on viral propagation. Adenoviruses hijack CRLs to negate these anti-viral p53 effects by degrading this master regulator.

Preventing the apoptotic-inducing ability of p53 however has the unwanted effect of potentially causing extensive DNA damage within infected cells. Adenoviruses (like Ad5) circumvent this by additionally targeting the MRE11 subunit of the host DNA damage sensing MRN complex (consisting of MRE11A-RAD50-NBS1) for degradation using the same E4ORF6–EB155K–CRL5 [26]. As mentioned, not all serotypes of adenoviruses target the same substrates, E4ORF6 from Ad12 dampens host DNA damage signaling by degrading TOPBP1 rather than the MRN [30,101]. This hijacking of different CRL family members to independently target DNA damage factors within the same pathway is a remarkable example of viral perturbation of the UPS. Further studies will hopefully reveal if this common theme exists within many viruses, perhaps as a redundancy mechanism by viruses to ensure blocking of a particular host response (in this case, the DNA damage response pathway). Targeting CRLs from the same archetype may be easier to achieve by viruses due to structural similarities between these ligases despite divergent substrate specificities. Finally, a number of other substrates of Ad5 E4orf6 and E1B55K have been identified including BLM (Bloom Helicase) [31], integrin α3 [32,102] and ATRX [102]. A clear picture of why this pair of viral proteins targets so many host factors and how these ubiquitination events are integrated is yet to emerge but these findings demonstrate the multi-faceted potential of viral-CRL hijacking.

The E7 protein of the Papillomavirus (HPV), a virus that plays a causative role in numerous mucosal cancers, also alters CRL activity. The HPV E7 protein is responsible for host cellular oncogenic transformation and binds an active form of CRL2 via a direct interaction with Elongin C. This leads to the ubiquitination and degradation of the host pRB tumor suppressor. [33]. Degradation of pRB in infected cells can derepress E2F dependent transcription. Over-expression or deregulation of E2F gene targets in turn abrogates cell cycle checkpoints assisting in the transforming ability of HPV. Interestingly, the E7 protein also binds to the proteasome [103] although this interaction is not required for pRb degradation [104]. This implicates a role for E7 in proteasomal targeting or chaperoning other substrates, which future studies will hopefully identify.

Kaposi’s sarcoma-associated herpesvirus (KSHV) is a Group-I rhadinovirus and the etiologic agent of several human malignancies that occur frequently in AIDS patients. KSHV proteins subvert many aspects of the host UPS (including CRLs) in several intricate cascades. Firstly, the KSHV latency-associated nuclear antigen (LANA) protein functions as a SOCS-box protein mimic within CRL5 complexes. The N-terminus of LANA contacts EloB/C while the C terminus interacts with Cul5 directly. This allows LANA to interact with host p53 and VHL (Von Hippel-Lindau Tumor Suppressor), driving their CRL5-dependent ubiquitination (Figure 3g) [34]. KSHV also targets the kinases responsible for degron phosphorylation upstream of CRL1. Specifically, KSHV v-cyclin interacts with host CDK6 leading to phosphorylation of p27 (a cell cycle inhibitor). This phosphorylation marks p27 for CRL1-dependent degradation [35]. In addition, KSHV LANA protein binds to and inhibits FBW7, the CRL1 SR inhibiting Intracellular Notch1 (ICN) poly-ubiquitination [34]. Degradation of both these substrates ultimately results in the proliferation of KSHV infected cells. Furthermore, KSHV activates NFκB signaling to both promote host cell proliferation and to promote latent infection by inducing the phosphorylation of IκBa driving its CRL1 dependent degradation. IκBa depletion results in the de-repression of NFκB signaling [34]. KSHV LANA also associates with glycogen synthase kinase-3 (GSK-3), inactivating it by a combination or mis-localization and mis-phosphorylation [36]. Inactivation of GSK-3 prevents phosphorylation of the degron on c-Myc required for its degradation by CRL1—further deregulating the growth of KSHV infected cells. Taken together, it is clear that KSHV re-directs a number of host CRLs to regulate key cellular pathways in proliferation and immunity to create a highly favorable cellular milieu for viral persistence.

The M-T5 protein of the Group I poxvirus Myxoma (a rabbit/hare virus) interacts with host CRL1 complexes. This interaction results in the ubiquitination and degradation of the cell cycle regulatory protein p27. Degradation of this CDK inhibitor overcomes the cell cycle arrest produced by infection and prevents infected cells from triggering their anti-viral responses, increasing infectivity [37].

CRL1 is a target of the Group-I Baculovirus Autographa Californica Multicapsid Nucleo-polyhedrovirus (AcMNPV). AcMNPV encodes a viral FBP (Lef7) that alters the host DNA damage response—often a requirement for the replication of large DNA baculoviruses. Although the direct host ubiquitinated target for Lef7 has not yet been found, it is thought to be linked to histone phosphorylation [38].

Murid herpesvirus-4 (MuHV-4), which causes mononucleosis in infected mice, expresses its ORF73 protein to target the NFκB family member RelA/p65 for proteasomal degradation. To do this ORF73, using a non-conventional SOCS-box motif, forms an active complex with CRL5. This inhibition of NFκB by RelA degradation is critical for the amplification of latent MuHV-4 and persistent infection within the host [39].

Avian adenovirus CELO (chicken embryo lethal orphan) expresses a GAM1 protein capable of recruiting both CRL2 and CRL5 complexes. The GAM1-CRL2/5 complexes subsequently target host SAE1 and SAE2 (Small Ubiquitin-like Modifier-1 (SUMO-1) Activating Enzyme Subunit 1/2) for proteasomal degradation. SAE1/2 normally form the heterodimeric E1 for the posttranslational modification SUMO-1. Although the downstream functional impact of this degradation is as yet unknown, it is likely to severely down-regulate SUMO-1 modifications within infected cells [40].

The polyomavirus SV40 (a simian/human virus) encodes the Large T antigen (LTAg)—a well-studied highly oncogenic viral protein. LTAg has recently been shown to bind to the carboxyl terminus of CRL7 (Figure 3h) [41]. This interaction impairs the ability of CRL7 to degrade its cognate substrate, the insulin receptor substrate 1 (IRS-1), a critical component of the insulin and insulin like growth factor 1 (IGF1) -signaling pathways. LTAg inhibition of CRL7 leads to the deregulation of the downstream effectors of IRS-1 (specifically the kinases Akt and Erk MAPK). Though the precise function of this is yet unknown, initial data suggests the SV40 LTAg-CRL7 interaction may alleviate the E3’s function as a tumor suppressor by depressing the downstream oncogenic IRS1 signaling pathways [42].

In addition to directly targeting CRL core complex components, Group-I viruses also target CRL regulators. A clear example of this is exhibited by Herpes Simplex Virus 1 (HSV1, the predominant cause of cold sores). HSV1 expresses its ICPo protein, which is capable of transactivating many host cellular genes. Although ICPo does not directly bind to CRLs, it is dynamically associated with proteasomes and enhances the ability of Cdc34 (ubiquitin-E2 for CRLs) to bind its E3 presumably increasing CRL complex activity to drive infectivity [43].

Perhaps, one of the most amazing examples of viral mimicry of host protein function is the recent discovery of how Epstein Barr virus (EBV, the causative agent of glandular fever) controls CRL activity. Gastadello and coworkers found the Epstein Barr BPLF1 protein was in fact a deneddylase with a similar enzymatic activity to the host CSN [45]. In infected cells, BPLF1 can presumably inactivate all CRLs resulting in the accumulation of CRL substrates. Specifically this results in chromatin licensing and DNA replication factor-1 (CDT1) accumulation (a “normal” CRL4 substrate). In the absence of CDT1 degradation, the cell cycle of infected cells becomes deregulated. The resulting S phase status of infected cells creates a highly favorable intracellular environment for viral replication. In addition to this apparently unique role in viral NEDD8 hijacking, EBV also plays a more canonical role in affecting CRLs. EBV genomes encode a BZLF protein that directly functions as a substrate adaptor protein in CRL2 complexes recruiting p53 for degradation. This may then allow for either the establishment of latent infection or in tumor formation in infected cells [44].

In summary, Group-I viruses co-opt CRLs with varied outcomes including both inhibition and promotion of host factor degradation by direct CRL interaction, as well as specifically targeting CRL regulators, with indirect but robust effects on CRL substrate ubiquitination.

### 5.2. Group-II (Single Stranded DNA Viruses)

Geminiviruses—members of Group-II, are plant viruses that cause extensive losses in crop production worldwide. Genomes of these viruses encode a C2 protein. It has been shown that in specific Geminiviruses (e.g., the Tomato Yellow Leaf Curl Sardinia Virus (TYLCSV)), C2 is capable of interacting with the catalytic subunit of the CSN complex (CSN5/Jab1). Expression of C2 in transgenic Arabidopsis plants decreases CSN activity and causes the accumulation of neddylated CRL1. As a result, pathways regulated by CRL1, including responses to hormones such as jasmonates, auxins, gibberellins, ethylene and abscisic acid, are altered in these plants [46]. Another Group II plant virus called faba bean necrotic yellows virus (FBNYV) expresses an F box protein (called CLINK) capable of binding SKP1 and intriguingly pRb [48]. However, no direct effect on CRL1 or host substrates has been demonstrated for CLINK; further insight into this protein will be highly intriguing.

Finally, infection by parvovirus minute virus of mice (MVM) causes CRL4 (with the SR CDT2) to drive degradation of the host protein p21—a cell cycle regulator [105]. Specifically, parvovirus infection recruits p21 to viral replication compartments within the nuclei, where it is ubiquitinated and degraded in a proliferating cell nuclear antigen (PCNA) dependent manner. The viral mechanism of this recruitment is yet to be resolved but p21 removal from cells is proposed to suppress the inhibition of host polymerase activity in infected cells driving viral DNA replication [47].

### 5.3. Group-III (Double Stranded RNA Viruses)

Group-III viruses include Reoviruses and Rotaviruses, which cause gastrointestinal infections. Rotavirus nonstructural protein-1 (NSP1) causes the degradation of the β-TRCP, (Beta-Transducin Repeat Containing E3 Ubiquitin Protein Ligase) an FBP constituent of CRL1 complexes [49]. Ordinarily, β-TRCP ubiquitinates IκBa, a protein that functions to inhibit the transcriptional activity of NFκB. However upon infection by Rotavirus, IκBa becomes stabilized preventing NFκB signaling and thus dampening the anti-viral interferon response in NSP1 expressing cells. Stabilization of IκBa and prevention of NFκB signaling is a clear example of how a virus inactivates host immune factors by hijacking the UPS degradation machinery.

### 5.4. Group-IV (Positive Sense ssRNA Viruses)

Hepatitis E is an RNA virus that causes hepatoma. In a similar manner to the abovementioned Rotavirus, the HepE ORF2 protein directly associates with β-TRCP [50]. This interaction also inhibits NFκB signaling and the interferon response negatively regulating the host immune system by preventing IκBa interaction with and degradation by CRL1.

Another member of this family is the Beet Necrotic Yellow Vein Virus (BNYVV)—a plant virus that infects sugar beet. This Group-III virus encodes a P25 protein that binds a Kelch-type FBP, which is involved in controlling plant resistance to infection through an unknown mechanism [51,52]. Interestingly, the P25 protein of Potato Virus X (PVX) causes the ubiquitination and degradation of host Argonaute1 (a central protein component in small RNA silencing pathways) in order to counteract the anti-viral effect of RNA silencing. No E3 has been identified as catalyzing the turnover of Argonaute1 in infected cells but it is possible that CRL1 is involved [53]. Another Group IV plant virus—polerovirus expresses a silencing suppressor protein (P0) that contains an Fbox motif. P0 binds to SKP1 in CRL1 complexes to target a yet undiscovered host factor involved in post-transcriptional gene silencing [54]. Together it is clear that Group IV viruses are known to date to target SRs of presumably active CRLs. Further work will most likely reveal more such interactions and discovery of the physiological substrates will determine why these viruses hijack CRLs *in vivo*.

### 5.5. Group-V (Negative Sense ssRNA Viruses)

As outlined above, in order for viruses to replicate unhindered by the host immune system the interferon response has to be deregulated. A key step in the interferon response is the activation of the transcription factors STATs (signal transducer and activator of transcription). The acquired immunity response activates just STAT1 whereas the innate immune response activates both STAT1 and STAT2. In cells stimulated with interferon STAT1 and STAT2 heterodimerize and interact with Interferon Regulatory Factor 9 (IRF9) to form a larger active transcription factor complex. This complex is capable of binding interferon stimulated response elements within the promoters of anti-viral genes driving their expression, an integral part of the host immune response. Group-V virus members have developed a mechanism co-opting CRLs to neutralize this host anti-viral response.

The paramyxovirus virus family contains many human and animal pathogens, particularly well-known are the Nipah (NiV), Hendra virus and Simian Virus 5 (SV5). Paramyxovirus V proteins are capable of binding the DDB1 adaptor subunits of CRL4 complexes [106]. Simian Virus 5 (SV5) expresses a V protein that can sequester cellular STAT2/STAT1 heterodimers driving the CRL4 dependent ubiquitination and degradation of STAT1 (Figure 3c). Without STAT1 the host interferon induced response to viral infection is greatly diminished [55,107,108]. A similar strategy is used by human parainfluenza virus 2 and mumps virus to degrade both STAT1 and STAT2 proteins—indicating that this is a common mechanism among a subset of viruses [56]. Further evidence of this theme is seen in Respiratory Syncytial Virus, (RSV)—the causative agent of pneumonia in infants. RSV encodes a non-structural protein (NS1) that forms a complex with CRL2 to also degrade STAT2 in infected cells [57]. The ultimate aim of this recurring viral bottleneck is to dampen host interferon signaling, reducing the ability of infected cells to counteract viral replication.

Viruses of this and other groups use several orthogonal approaches to down regulate the host anti-viral immune response. A clear illustration of this is within the livestock pathogen Rift Valley Fever virus, which expresses the non-structural, NSs protein. It has been recently shown that the direct binding of NSs to FBXO3 (an SR of CRL1) results in the degradation of a subunit of the TFIIH transcription factor [58,59]. This ultimately suppresses the transcriptional up-regulation of innate immunity factors within infected cells.

In summary, Group-V viruses have evolved several mechanisms to inactivate specific arms of host immunity. Future targeting of this pathway will hopefully prove promising as an anti-viral target.

### 5.6. Group-VI (ssRNA-RT Viruses {+ Strand or Sense} RNA with DNA Intermediate in Life-Cycle)

Human Immunodeficiency Virus *(*HIV) is a Group VI retrovirus and the causative agent of Acquired Immunodeficiency Syndrome (AIDS). HIV has a number of mechanisms by which it usurps CRL complexes to create a cellular environment conducive to viral replication: (1) HIV Viral infectivity factor (Vif)—binds primarily CRL5 (although it is also reported to bind CRL2) via a direct interaction with the adaptor proteins Elongin B (EloB) and Elongin C (EloC) as well as contacting Cul5 [60,92]. Simultaneously, Vif also hijacks a “co-transcription factor” CBF-β (core-binding factor subunit β) which together with CRL5 drives the Vif dependent poly-ubiquitination and degradation of A3 proteins (apolipoprotein B mRNA editing enzyme, catalytic polypeptide-like 3) (Figure 3d). Many diverse poly-ubiquitination sites are spread out within these substrates: A3F has ≥6 sites and A3G has ≥10 sites, in what appears to be a redundancy mechanism ensuring degradation [109]. In the absence of Vif, A3F and A3G are packaged into HIV virions within virus-producing cells. Upon subsequent infection of new target cells A3F and A3G negatively affect many steps of the HIV virus life cycle including: Hyper-mutation of viral DNA, inhibition of viral DNA synthesis, inhibition of viral integration and provirus formation. The combination of these adverse effects in the absence of Vif would render the produced HIV virions inactive [18]. Therefore, the clear physiological role of Vif to is counteract the anti-viral effects of A3’s primarily through the hijacking of a host CRL.

HIV Vpu—a transmembrane protein—assembles in a β-TRCP-containing SCF complex (Figure 3a). This complex targets the cytoplasmic domain of the CD4 receptor for poly-ubiquitination and degradation. Counter intuitively degrading the CD4 cell surface receptor (which is required for HIV entry into cells) prevents superinfection of the virus and allows for efficient viral release [110]. In addition, BST2 (Bone Marrow Cell Stromal Antigen 2, also called tetherin) is down regulated from the membrane in a Vpu dependent manner although the involvement of the UPS in this pathway is controversial and poorly understood [111]. Interestingly both HIV-2 Envelope protein and SIV negative-regulatory factor (Nef) also counteract BST2, though again the involvement of the host UPS is controversial [3].

The third HIV accessory protein known as Viral protein R (Vpr) binds to CRL4 complexes in HIV infected cells. Host CRL4 complexes are required for the recognition of UV-DNA damage in the absence of viral infection [21,112,113], and indeed Vpr expression arrests cell cycle progression at the G2 phase, in a similar manner to a DNA-damage related checkpoint [114]. In recent years, several putative substrates of Vpr containing CRL4 complexes have been identified, [61,115,116,117,118,119,120] with uracil DNA glycosylases (UNG2 and SMUG1) being the first recognized [118,119]. Yet, the significance of these Vpr interactions in the context of cell cycle arrest or viral infection is not clear. Recently, a new target for Vpr was described—the endonuclease Eme1-Mus81 [121]. This study demonstrated the CRL4 dependent ubiquitination of Mus81 in the presence of Vpr (Figure 3f). They also determined how Mus81 is targeted to Vpr-CRL4 by phosphorylation via the upstream Polo-like Kinase 1.

Many non-pandemic HIV strains and their simian counterparts (SIV) also contain the Vpr-like protein called Vpx. This small viral protein was also known to hijack CRL4 [122,123] and was recently shown to drive the degradation of the host SAM domain and HD domain-containing protein 1 (SAMHD1), a nucleotide triphospho-hydrolase that restricts lentiviral reverse transcriptional activity in myeloid cells [63] (Figure 3e). SAMHD1 has been described as suppressing the levels of dNTPs in several cell types [63] and CRL degradation of SAMHD1 may elevate dNTP levels above the threshold required for retroviral reverse transcription and subsequent infection to proceed. An alternate role for SAMHD1 has also been proposed whereby SAMHD1s ribonuclease activity is responsible for restricting viral replication by degrading viral RNA within infected cells [124].

In the case of lentiviruses, it seems that CRLs are not the only target among ubiquitin E3 ligases. Another large family of ligases is the tripartite motif (TRIM) family that includes more than 75 members. Many TRIM E3s restrict retroviruses, lentiviruses, and perhaps other viruses as well [125]. However, in the case of CRLs it is clear that they play a central role in the ability of retroviruses to inactivate specific host pathways and specifically counteract viral sensors within the host immune system. As discussed below, this makes CRL–viral interactions exciting targets for therapeutic interventions.

### 5.7. Group-VII (dsDNA-RT Viruses)

Hepatitis B (a causative agent of hepatocellular carcinoma) encodes the Hepatitis B virus X (HBx) protein that is often referred to as “enigmatic”. That said, it is clear that HBx plays an essential role in viral replication [62,64,65,126,127]. The HBx protein interacts with CRL4 through a specific contact with DDB1 [66] and requires an intact CRL4 to function. This implies the interaction is both functionally significant and dependent on E3 enzymatic activity. The HBx-CRL4 interaction results in host genome instability and cell death as well as an increase in viral replication and S phase arrest of infected cells [128]. This would imply that HBx either interferes with the CRL4 dependent degradation of host DNA damage/cell cycle factor(s) or directly drives the ubiquitination of a protein regulating one of these pathways. Unfortunately, neither function of HBx has been elucidated to date. However, HBx has been shown to stabilize protooncogene pituitary tumor-transforming gene 1 (PTTG1)—an inhibitor of p53. Whether this is CRL of even ubiquitin mediated requires further study [129].

## 6. Do Viral Proteins Targeting CRLs also Interact with the Proteasome?

It is unambiguous that many viral proteins interact with the host UPS including members of the CRL family. As outlined in this review a major theme of these interactions is in directing the degradation of host factors via poly-ubiquitination and the 26S proteasome. Viral interactions with the proteasome therefore appear in general to be indirect as most viral proteins (and especially those that interact with CRLs) divert the UPS prior to proteasomal targeting either at (or upstream of) the ubiquitin conjugation step. To date, few viruses have been shown to directly manipulate the proteasome. Some studies have begun to shed light on direct proteasome–virus interactions: The canonical (CRL independent) viral–proteasome interaction is observed with EBV and KSHV which both block antigen presentation in infected cells by inhibiting the 26S subunit of the proteasome using their EBNA and LANA proteins, respectively [34]. Proteasome inhibition is used as an “immune silencer” by such viruses preventing viral peptide presentation on the cell surface by the Major Histocompatibility Complex 1 (MHC1) and subsequent immune recognition by T lymphocytes.

Few direct viral–proteasome interactions have been documented: The Hepatitis HBx protein (mentioned above) is capable of binding directly to a subunit of the 19S proteasome [130] as well as to CRL4. As no substrate has been identified for this complex, it is plausible that HBx affects UPS steps downstream of CRL ubiquitin-transfer, perhaps at the step of substrate shuttling. A direct virus–proteasome connection has been found in the Group IV Potato Virus Y multifunctional protein helper component proteinase (HC-Pro) that interacts with three subunits of the plant 20S proteasome, though no function has been attributed to this interaction [131]. In another Group IV example, the SARS coronavirus nucleocapsid protein was reported to bind to the p42 subunit of the 26S proteasome, but again no function has been described [132].

HIV accessory factors also have tentative interactions with the proteasome. Vpr has been clearly shown to interact with CRL4 but also with RAD23—a ubiquitin binding protein that associates with the proteasome [133,134]. The Vpr-RAD23 interaction appears to still allow RAD23 to shuttle ubiquitinated cargo to the proteasome [134]. As it has recently been shown that a CRL4-Vpr complex can prematurely activate the SLX4/Mus81 host complex by ubiquitination [121], understanding the role of RAD23 in this process would be very informative. Finally, Herpes virus ICPo has been shown to interact with the proteasome as well as an E2, implicating another role for viral proteins in proteasome regulation.

As our knowledge of viral-UPS deepens, we will likely (a) discover more direct virus–proteasome connections and (b) determine the functions of such intriguing interactions—the mechanisms of which will lead to new and exciting biology. It is particularly likely that viral proteins have the ability to alter “global” host protein turnover rates directly through the proteasome to assist their pathogenesis by accelerating/inhibiting degradation of host and viral factors.

## 7. Could CRL Based Therapeutics Work as Anti-Virals?

As the UPS plays a central role in many different disease pathways including cancer, neurodegradative diseases and viral pathogenesis, therapeutically targeting its components is an exciting prospect. There has been significant success in targeting the proteasome as a cancer therapeutic with the small molecules Bortezomib and Carfilzomib [135]. However, as outlined here and by others [136], different viruses interact in very different ways with the host UPS and so broadly targeting the proteasome may not be a viable therapeutic option. Indeed, as some viral factors inhibit the degradation of host proteins while others initiate host factor degradation, proteasome inhibition may result in either viral suppression or a devastating increase in virulence. There are reports supporting this, in which Bortezomib treatment has increased the replication of mouse hepatitis and human RSV [137,138].

The prospect of successfully targeting an individual E3 ligase directly hijacked by a specific virus is much more appealing. A notable success again in the cancer field has been the use of Nutlins to target the MDM2/p53 axis [139]. Specifically for the case of CRLs, there is great enthusiasm in individually targeting members of this family [140,141,142]. As a proof of principle, the development of MLN4294 (a small molecule inhibitor of NAE1, see Figure 2) has shown that CRL activity can be inhibited and its impact is very promising in arresting the growth of many cancer cell lines as well as *in vivo* models of solid tumors [13]. MLN4294 is assumed to be a pan-CRL inhibitor targeting all members of the CRL family. In terms of anti-viral treatment, inhibition of CRL neddylation by MLN4294 has shown efficacy in HIV infected cells presumably by preventing (at a minimum) Vif, Vpu and Vpr from degrading their host targets [98,143].

A further and potentially more fruitful area of pursuit will be targeting individual CRLs (e.g., CRL5/Vif inhibition as a HIV therapeutic) or even targeting CRL regulators (e.g., the CSN). Interactions between cellular restriction factors (such as A3G and SAMHD1), their cognate viral proteins (e.g., HIV Vif and SIV Vpx) and the host CRL (CRL5 and CRL4) are enticing targets for anti-viral drug development as augmenting the restriction factor (or inhibiting the viral protein) would allow the hosts’ immune system to control and counteract viral infection. Recent structural elucidations at the atomic level of these host CRL complexes containing viral factors have provided valuable information in identifying targetable interfaces [92]. Although these studies are in their infancy, the concept of blocking host–viral interactions (rather than the traditionally targeted viral–viral interactions) was successfully proven in the case of Maraviroc [144]. This small molecule binds to the cell surface receptor C-C chemokine receptor type 5 (CCR5), a co-receptor required for HIV binding to the membrane—blocking its interaction with HIV GP120 thereby preventing HIV entry into cells. Strides have been made in the direction of targeting the CRL-host-viral axis in an analogous fashion; small molecules capable of augmenting A3 levels even in the presence of Vif have been developed [145,146], although their precise mechanism of targeting is as yet undefined. Undoubtedly, future studies will both improve on the range of CRLs targeted and their efficacy. The major concern, however, is that in cancer cells aberrant CRL activity can remove anti-proliferative factors/signals which therapeutic inhibition can slow/reverse. With viral infection pharmacologically altering a host, E3 that simultaneously performs virus-independent functions in many cell types may not prove to be feasible.

## 8. How to Identify CRL Substrates?

Determining the identity of host factors degraded by CRLs in both the presence and absence of viral proteins is essential in understanding both the host UPS and viral pathogenesis. To date, substrate identification has been hampered by two unavoidable biological principals: (1) substrate–E3 interactions are generally transient in nature making it difficult to “trap” a substrate-loaded CRL complex; (2) Substrates are turned over quickly and often exist as only a small proportion of the total protein within a cell and are therefore present at very low levels of abundance. Several experimental approaches have been developed to identify novel substrates of E3s in general—as well as those specifically degraded by CRLs [147]. A combination of genetic (global protein stability (GPS)) and proteomic (QUAINT: quantitative ubiquitination interrogation) methods have already identified hundreds of E3 targets [148]. In addition, technologies specifically addressing the above two shortcomings have been elegantly addressed: (A) Substrate-ligase trapping technology has been developed using either a ubiquitin binding domain fused to an SR [149] or by mutating the SR [150,151]—increasing the binding affinity of the substrate to the SR eliminating the issue of transient substrate-CRL binding. Substrates can then be identified by standard immuno-precipitation mass spectrometry (IPMS) approaches. (B) To overcome substrate abundance issues, ubiquitin remnant profiling has proven very successful. This works by immuno-purifying the unique di-glycine motif left after tryptic digests of ubiquitin-conjugated peptides. The resulting fold-enrichment from the purification allows identification of low abundance targets although does not directly determine the responsible E3.

The approaches mentioned above will prove powerful in identifying large numbers of CRL substrates, however, it is informative to note how the major CRL-viral–substrate interactions were found to date. Only through the use of elegant phenotype driven approaches have *bona fide* viral-CRL substrates been identified. Initially many approaches were genetic (CRL5-Vif-A3 and CRL1-Vpu-BST2 discoveries) although proteomic approaches have also more recently been successful (CRL4-Vpx-SAMHD; CRL4-Vpr-MUS81). These and further studies aimed at uncovering virally driven CRL degradation will undoubtedly reveal novel aspects of host immunity.

However, it is clear that many of the studies reviewed here highlight that care should be taken in interpreting CRL substrate interactions in virally immortalized cell lines (such as 293T and COS-7 cells). Indeed, it seems likely that the CRL-UPS axis is perturbed in all virally transformed cells. Carrying out such studies in either several cell lines or in viral factor free cell lines should be a priority.

A recent study determined the global landscape of HIV-1 human protein–protein interactions through the expression of individual, affinity tagged HIV-1 proteins in two different human cells, followed by affinity purification and extensive mass spectrometry [98]. The resulting network of interactions show that many HIV-1 proteins interact with a broad range of enzymes and regulators of the host UPS. Similar studies of other viruses in combination with the methodologies discussed above will reveal new viral–CRL connections. Furthermore, the use of such methods in combination with specific CRL inhibitors (when developed) will be highly informative of direct substrate targets and may provide a highly specific therapeutic opportunity.

## 9. What Open Questions Remain Regarding CRLs in Viral Infections?

The past decade has seen a huge expansion in our knowledge of CRL biology. Specifically, since the discovery of the Vif-CRL5-A3G axis (Figure 3d) [152], the roles of CRLs (and their regulators) in viral infection have uncovered a broad range of novel molecular mechanisms and pathways. It is clear that this field will continue to expand, particularly as a result of vast improvements in sequencing efforts identifying new viruses combined with the development of high throughput proteomic methods (as mentioned above) capable of deciphering hundreds of host–viral interactions in a single study [60,98,153]. Although we have mostly discussed viral subversion of the UPS resulting in the removal of an anti-viral host factor—it is important to note that there are other outcomes for UPS–viral interactions. For example, many viral proteins themselves get ubiquitinated, yet the E3s responsible for these are largely unknown [9]. To illustrate this example, CRL5 has been shown to ubiquitinate Vif. Perhaps, this form of “auto-ubiquitination” is due to aberrant CRL regulator interaction or it points towards an anti-viral role for host CRLs in removing viral proteins from infected cells. Further work is needed to test this hypothesis.

Among the CRLs targeted by viruses, two major themes are evident: (1) FBPs are targeted in many species by many viruses and (2) viruses themselves encode FBPs. The range of substrates targeted by these FBPs is not well catalogued. Identifying these substrates will be very important in looking for common viral strategies used to hijack the UPS and in identifying therapeutically targetable CRL–substrate pairs. Understanding why viral hijacking strategies converge on FBP proteins to alter host UPS pathways will be fascinating.

Furthermore, as well as identifying potential host and viral ubiquitination targets, the type of ubiquitination linkage will be important in understanding the mechanistic outcomes. Most studies to date focus on the degradative ubiquitination but it is likely that many host–viral ubiquitination events are regulatory (and non-degradative) in nature—focusing on this subset will be very informative.

Another level of understanding will also come with solving the structural mechanisms of virus–CRL interactions. Although recent structures have helped initiate these studies, very little is known about the stoichiometry, orientation and subunit contacts with viral proteins in CRL complexes with a few exceptions (such as the SV5-CRL4 interaction [56]).

At a more functional level, how virus–CRL complexes integrate to modify the cellular environment is also not well deciphered. An example again is highlighted by the differential hijacking of adenovirus’ proteins of a number of CRL family members to target related host factors. This may be a general viral principle where perhaps there is not just one target for a host–viral CRL complex and moreover the same viral protein from different strains may have diverged targets. If so, then care must be taken in attributing a single substrate to an individual viral–CRL interaction or conformation.

## 10. Conclusions

In conclusion, the field has demonstrated that viral proteins: (i) Serve as substrates themselves; (ii) redirect active host CRLs to degrade otherwise long-lived host proteins; (iii) are capable of inhibiting CRL ubiquitination activity; and (iv) indirectly affect CRLs by interacting with and modulating their regulators. These diverse and complex interactions all function to tip the balance of the host intracellular environment to drive viral pathogenesis. In many ways, uncovering these mechanisms has informed us as much about our own immune system as it has about infectious pathways. Our understanding of the intricacy of CRL biology will continue to expand as more such elegant mechanisms come to light.

## Abbreviations

A3F/G: Apolipoprotein B mRNA editing enzyme, catalytic polypeptide-like 3 F/G
AcMNPV: Autographa californica multicapsid nucleo-polyhedrovirus
AIDS: Acquired immunodeficiency syndrome
ATRX: X-linked α-thalassaemia retardation syndrome protein
β-TRCP: Beta-transducin repeat containing E3 ubiquitin protein ligase
BLM: Bloom helicase
BNYVV: Beet benyvirus necrotic yellow vein virus
BST2: Bone marrow cell stromal antigen 2
CAND1: Cullin-associated NEDD8-dissociated protein 1
CBF-β: Core-binding factor subunit beta
CDT1: Chromatin licensing and DNA replication factor-1
CDT2: Chromatin licensing and DNA replication factor-2
CELO: Chicken embryo lethal orphan virus
CSN: COP9 signalosome
CSA1: Cockayne syndrome A
CRL: Cullin-RING E3 ligase
DCAF: DDB1/Cul4A associated factor
DCN1: Defective in cullin neddylation-1
DDA1: DET1- and DDB1-associated protein 1
DDB1: DNA damage binding protein-1
DDB2: DNA damage binding protein-2
EBV: Epstein Barr virus
EloB: Elongin B
EloC: Elongin C
FBNYV: Faba bean necrotic yellows virus
FBP: F-box protein
GLMN: Glomulin
GPS: Global protein stability
GSK-3: Glycogen synthase kinase-3
Hbx: Hepatitis B virus X *protein*
HepB/E: Hepatitis B/E virus
HIV-1: Human immunodeficiency virus-1
HPV: Human papillomavirus virus
HSV1: Herpes simplex virus 1
Human RSV: Human respiratory syncytial virus
ICN: Intracellular notch1
IκBa: NF-kappa-B inhibitor alpha-like
IPMS: Immuno-precipitation mass spectrometry
IRF9: Interferon regulatory factor 9
KSHV: Kaposi sarcoma-associated herpesvirus
LANA: Latency-associated nuclear antigen
LTAg: Large T antigen
MAPK: Mitogen-activated protein kinase
MHC1: Major histocompatibility complex 1
MPN: Mpr1p and PAD1p N-terminal)/JAMM (JAB1/MPN/Mov34 metalloenzyme)
MRN: MRE11A-RAD50-NBS1 complex
MuHV-4: Murid herpesvirus-4
MVM: Minute virus of mice
NAE1: NEDD8 activating enzyme-1
NEDD8: Neural precursor cell expressed developmentally down-regulated protein 8
Nef: Negative regulatory factor
NFκB: Nuclear factor kappa-light-chain-enhancer of activated B cells
NHEJ: Non-homologous end-joining
NiV: Nipah virus
NS1: RSV non-structural protein 1
NSP1: Rotavirus nonstructural protein-1
NSs: Rift valley fever virus non-structural protein
PCNA: Proliferating cell nuclear antigen
pRB: Phosphorylated retinoblastoma protein
PTTG1: Protooncogene pituitary tumor-transforming gene 1
QUAINT: Quantitative ubiquitination interrogation
ROC1/Rbx1: Regulator of cullins/RING box protein-1
ROC2/Rbx2: Regulator of cullins/RING box protein-2
SAMHD1: SAM domain and HD domain-containing protein 1
SCF: Skp1-Cul1-F-box
SIV: Simian immunodeficiency virus
SKP1: S-phase kinase-associated protein-1
SOCS: Suppressor of cytokine signaling
SMUG1: Single-strand-selective monofunctional uracil-DNA glycosylase 1
SR: Substrate receptor
STAT: Signal transducer and activator of transcription
SUMO-1: Small ubiquitin-like modifier-1
SV40: Simian Virus 40
SV5: Simian Virus 5
TFIIB: Transciption factor IIB
TOPBP1: Topoisomerase (DNA) II binding protein
TYLCSV: Tomato yellow leaf curl sardinia virus
Ube2f: Ubiquitin-conjugating enzyme E2M
Ube2n: Ubiquitin-conjugating enzyme E2N
UNG2: Uracil N glycosylase 2
UPS: Ubiquitin-proteasome system
VACV: Vaccinia virus
VHL: Von Hippel-Lindau tumor suppressor
Vif: Viral infectivity factor
Vpr: Viral Protein R
Vpu: Viral Protein U
Vpx: Viral Protein X
